# Principles of Entrainment: Diagnostic Utility for Supraventricular Tachycardia

**Published:** 2008-02-01

**Authors:** George D Veenhuyzen, F Russell Quinn

**Affiliations:** Libin Cardiovascular Institute of Alberta, University of Calgary and Calgary Health Region, Foothills Medical Centre, Calgary, Alberta, Canada

**Keywords:** Entrainment, supraventricular, diagnosis, orthodromic, antidromic

## Abstract

Entrainment is an important pacing maneuver that can be used to identify reentry as a tachycardia mechanism and define components of the circuit. This review examines how principles of entrainment can be used to arrive at a firm supraventricular tachycardia diagnosis using a simple algorithm and builds a foundation for the application of entrainment to more complex or unknown circuits.

Entrainment is a pacing maneuver that has traditionally been applied during macroreentrant tachyarrhythmias to determine whether a pacing site is a part of the circuit. This information is in turn used to identify critical channels that may be targeted for ablation. Accordingly, entrainment has received much attention in the exploration of scar related ventricular and atrial tachycardia (AT) circuits, previously reviewed [1]. In contrast, the critical area that is targeted in supraventricular tachycardia (SVT) is often clear (the focus of an atrial tachycardia, the operative accessory pathway (AP), or the slow atrioventricular node pathway) though the correct SVT mechanism may not be. This review will focus on using entrainment to rapidly determine the correct SVT diagnosis by examining familiar SVT mechanisms such as AT, atrioventricular node reentry tachycardia (AVNRT), and orthodromic atrioventricular reciprocating tachycardia (AVRT). This information should be of practical value while building a foundation for the application of entrainment to more complex arrhythmias.

## What is entrainment?

To answer this question, we will consider a common and simple reentry circuit: orthodromic atrioventricular reciprocating tachycardia (AVRT). It is widely taught that a His-refractory ventricular premature beat (VPB) can advance atrial timing during AVRT. The VPB depolarizes the ventricles earlier than the tachycardia wavefront would have, and this in turn advances atrial activation so that the AVRT circuit is reset. If this rather basic electrophysiologic concept is well understood, then entrainment, which is nothing more than the *continual resetting* of such a circuit by a series of consecutive VPB's (ie. a pacing train slightly faster than the tachycardia), should be easily understood also.

***Teaching Point #1:*** *Entrainment is the continual or repeated resetting of a reentrant tachycardia by each of a series of consecutive beats of a pacing train.*

**Figure 1:**
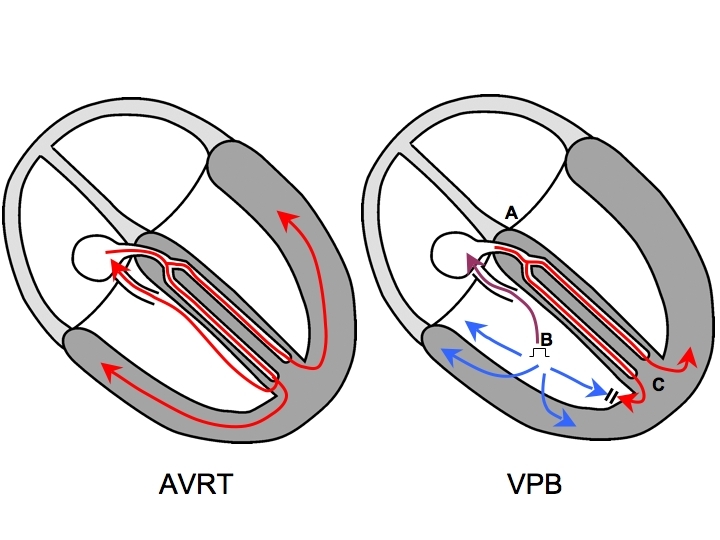
An orthodromic AVRT circuit employing a septal AP is depicted on the left. On the right, as the AVRT wavefront passes through the His bundle (A), a VPB is delivered (B). As the AVRT wavefront propagates from (A) to (C), the stimulated wavefront also propagates and a portion will travel in the same direction that the tachycardia would (orthodromic wavefront, purple) to advance atrial timing and reset the next beat of tachycardia. Another part of the stimulated wavefront will travel opposite to the direction that the tachycardia would (antidromic wavefront, blue) to collide with the AVRT wavefront in ventricular muscle. Because 2 wavefronts contribute to ventricular depolarization, there is fusion. If the VPB is delivered early enough, the collision between the antidromic wavefront and the AVRT wavefront could occur in the AV conduction system, so a fused QRS complex would not be observed (the QRS morphology would be that of a fully-paced beat). AVRT=atrioventricular reciprocating tachycardia; AP=accessory pathway; VPB=ventricular premature beat.

Let us consider that familiar VPB in more detail (Figure 1). In saying that the VPB is His-refractory, we must be mindful that it occurs as the tachycardia wavefront is approaching or passing through the His bundle (so that the wavefront from the VPB could not possibly conduct up the His bundle retrogradely due to refractoriness). At that time, there are 2 wavefronts simultaneously competing to depolarize the ventricles: the stimulated wavefront (which has a head start that is equal to its degree of prematurity) and the tachycardia wavefront that immediately preceded the VPB. The latter is termed the *orthodromic wavefront from the preceding beat*.  A portion of the stimulated wavefront traverses the AP to advance the subsequent atrial activation and is called the *orthodromic stimulated wavefront* (orthodromic because it travels in the same direction as that which the tachycardia wavefront would travel in). There is also a portion of the stimulated wavefront (the *antidromic stimulated wavefront*) that eventually collides against the orthodromic wavefront from the preceding beat (antidromic because it travels in the *opposite direction* to that which the tachycardia wavefront would travel in). The collision between the antidromic stimulated wavefront and the orthodromic wavefront from the preceding beat may occur in ventricular myocardium or in the AV conduction system, depending on timing (which, as we shall see later on, is influenced by the pacing site). If it occurs in ventricular myocardium, then the VPB will be a fusion beat; its morphology will differ from that of the tachycardia (because it is partly paced) and from that of a paced beat (because it is partly activated via the AV conduction system). In this case, a fused VPB morphology is also proof that the VPB was His-refractory.

**Figure 2:**
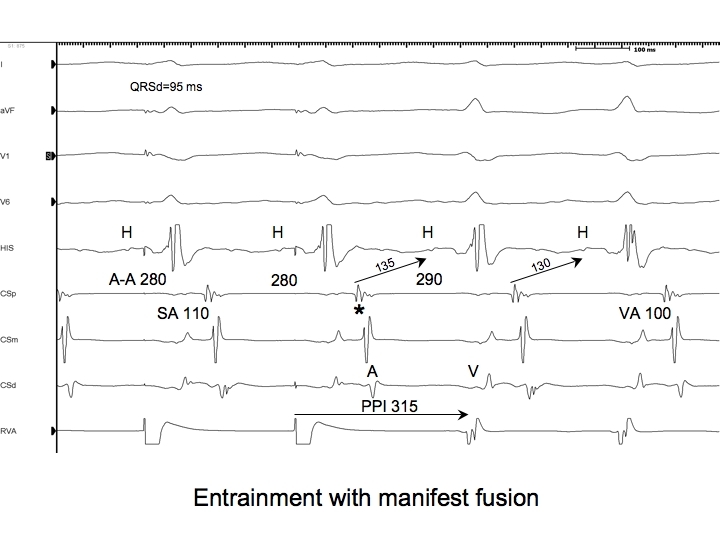
The last 2 beats of a pacing train at a CL of 280 ms from the RV apex, followed by ongoing orthodromic AVRT employing a posteroseptal AP with a TCL of 290 ms. Shown are surface ECG leads I,aVF, V1, and V6 and intracardiac recordings from catheters at the His bundle region (HIS), proximal coronary sinus (CSp), mid CS (CSm), distal CS (CSd) and right ventricular apex (RVA). The atria are accelerated to the pacing CL, and the response after pacing including the last accelerated atrial electrogram is A-V. During pacing the QRS complexes are too narrow to be purely paced beats (QRS duration=95ms). They result from fusion between the stimulated antidromic wavefront and the orthodromic wavefront from the previous beat. In addition to a fused QRS complex, there is an orthodromically captured far field His bundle potential present during pacing, which is further evidence of fusion. Note that the last entrained atrial beat (*) returns to the ventricles without evidence of fusion: the last beat is entrained but not fused. The AH interval on the first return beat is 5 ms longer than the AH interval of the tachycardia due to decremental conduction through the AVN at the shorter pacing CL. Therefore, the PPI-TCL difference (25 ms) must be corrected by 5 ms (20 ms). The SA-VA interval difference is 10 ms. S=stimulus; A=atrium; V=ventricle; H=His; PPI=post pacing interval; TCL=tachycardia cycle length; AP=accessory pathway; AVRT=atrioventricular reciprocating tachycardia. All measurements are rounded off to the nearest 5 ms.

Now consider orthodromic AVRT with a cycle length (CL) of 290 ms (Figure 2). During AVRT, a 12 beat pacing train from the right ventricular apex (RVA) is delivered at a CL of 280 ms after which, AVRT continues. During overdrive pacing, the atria are accelerated to a cycle length of 280 ms, and the QRS complexes are fused. The AVRT circuit has been successfully entrained: each paced beat accelerated the atria to the pacing CL (stimulated orthodromic wavefront) and simultaneously collided with the preceding orthodromic wavefront to produce a fused QRS complex. The last stimulated orthodromic wavefront continues around the circuit, traversing the AP, depolarizing the atria and returning to the ventricles over the normal AV conduction system (without fusion because there is no new stimulated antidromic wavefront to collide with) so that the tachycardia can continue. These features meet the first criterion for entrainment: there is constant fusion at a constant pacing CL and the last beat is entrained but not fused [2]. The presence of fusion is the key element that proves the tachycardia was entrained, so this is called *manifest entrainment.*

***Teaching Point #2:*** * Entrainment with fusion = manifest entrainment*

Isorhythmic dissociation of the pacing train from the tachycardia can mimic constant fusion, so pacing rates that are clearly faster than the tachycardia are required. Having said that, a pacing CL that is 10-20 ms shorter than the tachycardia CL is usually sufficient. It is important to show that the tachycardia resumes after pacing at a longer CL than the pacing CL.

Let us consider that familiar VPB once again. The ability of a His-refractory VPB to advance atrial timing of an SVT is proof of the presence of an AP. If the atrial activation sequence does not change, then the AP is almost certainly participating in the SVT mechanism, and the diagnosis is AVRT. While this observation does not exclude an AT originating close to the atrial insertion of a bystander concealed AP, this situation is rare enough that for all intents and purposes, it is practically ignored.

The implications of manifest entrainment of an SVT by ventricular pacing are similar, yet even more robust. Manifest entrainment is proof that the ventricle is a part of the SVT circuit because fusion is due to collision of the antidromic stimulated wavefront with the orthodromic wavefront from the preceding beat occurring within ventricular myocardium. A diagnosis of AVNRT or AT is impossible, because the stimulated wavefront could not possibly reach the AV node (AVN) or the atrium,^A^  fusion is proof that the His-Purkinje network would be refractory to retrograde conduction. Another way of saying this is as follows: since the orthodromic wavefront from the preceding beat must be using the AV conduction system to reach the ventricles and fuse with the stimulated antidromic wavefront, the orthodromic wavefront must be using an AP to conduct to the atria and continuously reset (entrain) the tachycardia. While single beat resetting with a His-refractory VPB identifies a circuit that can sustain a single echo of AVRT, entrainment with constant fusion identifies a sustainable AVRT circuit (that could otherwise sustain AVRT at the pacing CL).

***Teaching Point #3:*** * Manifest entrainment of SVT by ventricular pacing is proof that AVRT is present.*

**Figure 3:**
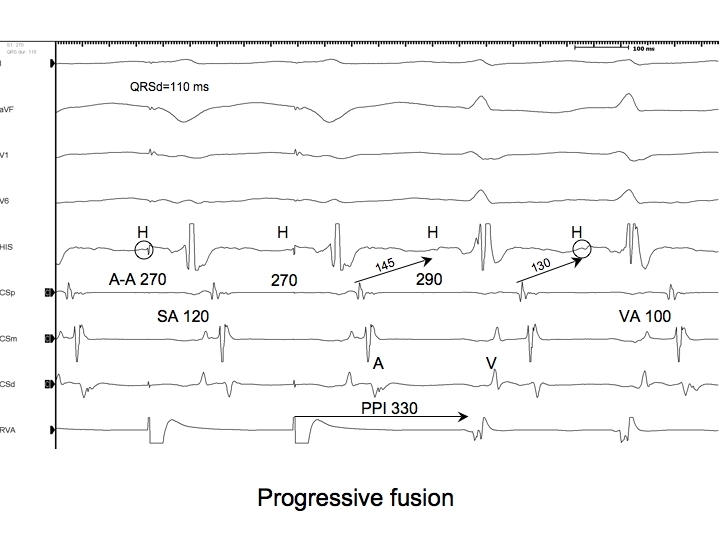
The last 2 beats of a pacing train at a CL of 270 ms from the RV apex, followed by ongoing orthodromic AVRT employing a posteroseptal AP with a TCL of 290 ms. Shown are surface ECG leads I,aVF, V1, and V6 and intracardiac recordings from catheters at the His bundle region (HIS), proximal coronary sinus (CSp), mid CS (CSm), distal CS (CSd) and right ventricular apex (RVA). The atria are accelerated to the pacing CL. The QRS complexes during pacing are still too narrow to result purely from pacing, yet they are wider and visibly different from those seen during entrainment at a CL of 280 ms. A different degree of constant fusion is present at the shorter CL because the stimulated antidromic wavefront can depolarize more ventricular muscle. At this shorter pacing CL, the first return AH interval exceeds the AH interval during tachycardia by 15 ms. Thus, PPI-TCL=40 ms, cPPI-TCL=25 ms, and SA-VA=20 ms. S=stimulus; A=atrium; V=ventricle; H=His; PPI=post pacing interval; TCL=tachycardia cycle length; AP=accessory pathway; AVRT=atrioventricular reciprocating tachycardia. All measurements are rounded off to the nearest 5 ms.

Let us again consider the AVRT circuit described above with a CL of 290 ms. At first, it was entrained by overdrive ventricular pacing at a CL of 280 ms, and the QRS complex during pacing was fused. Next, overdrive ventricular pacing is initiated at a CL of 270 ms (Figure 3). During pacing, the QRS complex is fused, and the tachycardia continues when pacing is stopped: that tachycardia has again been entrained. Because the pacing CL is shorter, the antidromic stimulated wavefront will start depolarizing the ventricles earlier relative to the orthodromic wavefront from the preceding beat, and will therefore be able to depolarize more of the ventricles. Hence, the fused QRS complex will resemble that of a paced beat more than the fused QRS complex that resulted from entrainment at a CL of 280 ms. Thus, the degree of fusion is dependent on the pacing CL.

***Teaching Point #4:*** * Progressive fusion is the second criterion for entrainment: at a constant CL, the degree of fusion is constant (part of criterion 1), while at a shorter CL, there is a different degree of fusion (that is constant at the shorter CL).*

## Atrioventricular Node reentry Tachycardia

**Figure 4:**
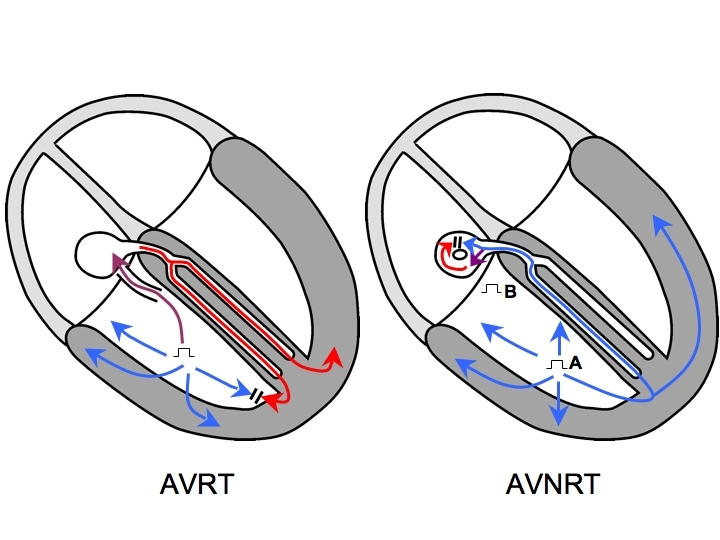
Entrainment of orthodromic AVRT is depicted in the left panel showing fusion due to wavefront collision in ventricular muscle (manifest entrainment). Entrainment of AVNRT is depicted in the right panel: in order to reach the AVN, the antidromic wavefront (blue) must be delivered prior to His bundle refractoriness, which is so early that it depolarizes all of the ventricular muscle, and the QRS complex morphology is that of a paced beat. Collision of the stimulated antidromic wavefront with the orthodromic wavefront from the previous beat occurs entirely within the AV node, where no recordings are available, so evidence of fusion is unavailable (concealed entrainment), regardless of the type of AVNRT circuit. Also, note that the pacing site is close to or part of the AVRT circuit on the left, but far from the AVNRT circuit on the right. Hence, the PPI-TCL difference is well suited for differentiating AVRT from AVNRT. Furthermore, the PPI-TCL and SA-VA differences ought to be longer after entraining AVNRT by pacing from a basal site (B) than an apical one (A). AVRT=atrioventricular reciprocating tachycardia; AVN=atrioventricular node AVNRT= AVN reentry tachycardia.

Let us now consider the most commonly encountered SVT in the electrophysiology laboratory: AVNRT. Entrainment of any version of AVNRT by overdrive ventricular pacing is certainly possible, though as we shall see, very difficult to prove. For ventricular pacing to entrain AVNRT, the stimulated wavefront must occur early enough that it can travel retrogradely up the His-Purkinje network and reach the circuit in the AVN. If at that point, an excitable gap is present, a portion of that wavefront (the orthodromic wavefront) will enter and advance the AVNRT circuit (and atrial activation) while another portion of that wavefront (the antidromic wavefront) will collide with the orthodromic wavefront from the preceding beat inside the AVN (Figure 4). Importantly, fusion cannot be present because the antidromic wavefront and the orthodromic wavefront from the preceding beat collide in AV nodal tissue: the QRS morphology during pacing must be entirely that of a paced beat. If the AVNRT was entrained, then AVNRT will continue when pacing is stopped. Because the QRS complex during entrainment is not fused, proof of entrainment is not available, so this is called concealed entrainment.

***Teaching Point #5:*** * While AVNRT of any kind can be entrained by ventricular pacing, QRS complex fusion is impossible (concealed entrainment).*

## Atrial Tachycardia

Finally, let us consider what would happen if overdrive ventricular pacing was introduced during AT. This has been examined in an elegant study [3] where patients with spontaneous AT or a simulated AT (produced by rapid atrial pacing) were included, along with patients with AVN dependent forms of SVT (AVNRT and orthodromic AVRT). Only cases where ventricular pacing accelerated the atria to the pacing CL followed by resumption of the tachycardia upon cessation of pacing were included in the study. The response immediately after the last paced QRS complex was classified as atrial-ventricular (A-V, Figure 2 and3) or atrial-atrial-ventricular (A-A-V). In every case of AT, the response after pacing was A-A-V, while in every case of AVNRT or AVRT, it was A-V (as we have seen above). This held true even when atrial tachycardia was simulated by atrial pacing in patients with dual AVN physiology or concealed APs. It seems that the stimulated wavefront penetrates both routes of retrograde conduction so that neither are available to echo that impulse back to the ventricles before conducting the first beat of AT to the ventricles. Thus, when overdrive pacing accelerates the atria, a post overdrive ventricular pacing response of A-A-V is specific for AT.

Unfortunately, a common finding during overdrive pacing of AT is that the atrium is not accelerated, and the ventricles are simply dissociated from the tachycardia [4,5]. Nevertheless, this observation is still diagnostically useful in that it allows AVRT to be excluded. While this response is most commonly encountered with AT, AVNRT is still possible, and further diagnostic information would be required to differentiate between these 2 SVT mechanisms.

***Teaching Point #6:*** * when overdrive ventricular pacing accelerates the atria, a post pacing response of A-A-V is specific for AT.*

***Teaching Point #7:*** * when overdrive ventricular pacing fails to accelerate the atria, a diagnosis of AT is most likely, but AVNRT must still be excluded.*

In determining the atrial - ventricular response relationship after overdrive ventricular pacing, it is of key importance to identify the last accelerated atrial electrogram and count it as the first atrial response after pacing. Failure to do so could result in a pseudo A-A-V response, as can be seen whenever the SVT utilizes anything other than a conventional AP or a fast AVN pathway for ventriculoatrial (VA) conduction (eg. orthodromic AVRT employing a slowly conducting AP, fast-slow AVNRT, slow-slow AVNRT) [6]. Another common pitfall in the interpretation of this response can be avoided by identifying the response as atrial-His (AH) versus atrial-atrial-His  (A-A-H) rather than A-V versus A-A-V [7]. These and other pitfalls are reviewed in the original study [3].

## Entrainment by ventricular pacing as a diagnostic tool

At this point, using overdrive ventricular pacing should seem like an ideal tool for SVT diagnosis:  (i) if the post pacing response is A-A-V, the diagnosis is AT, while if it is A-V, the diagnosis is either AVNRT or AVRT, and (iia) QRS complex fusion should identify AVRT, while (iib) the absence of fusion should identify AVNRT. Unfortunately, things are not that simple. While (i) is true, the problem with (iia) and (iib) is that, like most features used for SVT diagnosis [4], QRS fusion has traditionally been insensitive. That is, when overdrive ventricular pacing is performed during AVRT as it is most commonly performed - from the RVA - fusion is often not present. In fact, in early studies of entrainment, where orthodromic AVRT circuits employing left sided AP's (which account for about half of all cases of AVRT) were entrained by overdrive pacing from the RVA, it was thought that fusion could not be demonstrated [8]. Importantly, others were subsequently able to show that when the ventricular pacing site was located closer to the AP, fusion was demonstrable [9,10]. In these studies, manifest entrainment occurred after overdrive pacing from the RVA in 13 of 14 patients with septal AP's [10] and after LV pacing in 6 of 6 patients with left sided AP's (and 0 of 6 patients after overdrive pacing from the RVA) [9]. Despite these studies, fusion during entrainment of orthodromic AVRT has not received much attention as a diagnostic tool.

**Figure 5:**
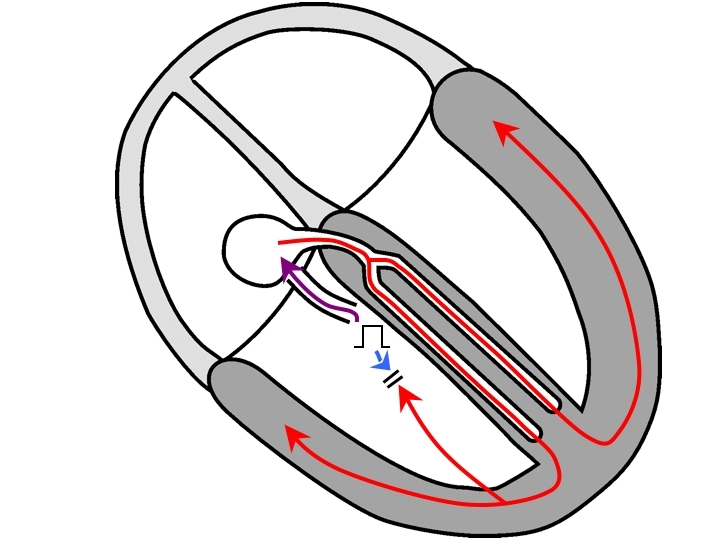
A schematic of entrainment with concealed fusion. The orthodromic wavefront from the previous beat (red) depolarizes virtually all of the ventricular muscle so that the QRS complex during entrainment is virtually identical to that of the tachycardia. The stimulated antidromic wavefront depolarizes very little ventricular muscle (blue), while the stimulated orthodromic wavefront (purple) continually accelerates the atria to the pacing cycle length and resets the tachycardia.

**Figure 6:**
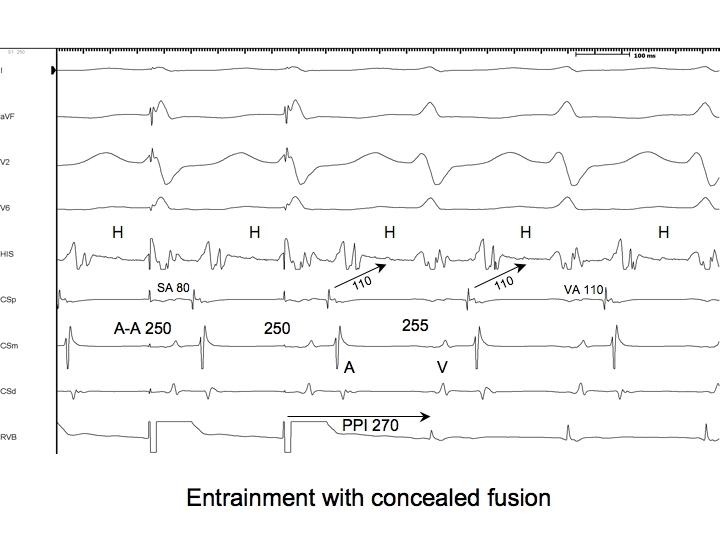
The last 2 beats of a pacing train at a CL of 250 ms from the RV base, followed by ongoing orthodromic AVRT employing a posteroseptal AP now with a TCL of 255 ms. Shown are surface ECG leads I,aVF, V1, and V6 and intracardiac recordings from catheters at the His bundle region (HIS), proximal coronary sinus (CSp), mid CS (CSm), distal CS (CSd) and right ventricular base (RVB). The atria are accelerated to the pacing CL. The QRS complexes during pacing are virtually identical to those of the tachycardia, though they are disturbed by the pacing stimulus, which actually occurs after the onset of the QRS complexes. Orthodromically captured His bundle potentials are present during pacing with an identical HV interval as during tachycardia. The only evidence that this represents entrainment rather than isorhythmic dissociation of the pacing train from the tachycardia is the constant acceleration of the atria to the pacing CL followed by immediate slowing of the tachycardia after the last entrained atrial electrogram. Because the pacing CL is so similar to the TCL, the first return AH interval is the same as the AH interval during tachycardia (no significant slowing through the AVN). PPI-TCL=15 ms. SA-VA=-30 ms. These values indicate that the pacing site is in the circuit and close to the AP. S=stimulus; A=atrium; V=ventricle; H=His; PPI=post-pacing interval; TCL=tachycardia cycle length; AP=accessory pathway; AVRT=atrioventricular reciprocating tachycardia; AVN=atrioventricular node. All measurements are rounded off to the nearest 5 ms.

The pacing site most likely to produce fusion is that from which the stimulated antidromic wavefront depolarizes the least amount of ventricular muscle, thereby allowing the orthodromic wavefront from the preceding beat to depolarize as much ventricular muscle as possible before the two wavefronts collide (Figure 5).  Because the orthodromic wavefront from the preceding beat must exit the Purkinje network to depolarize ventricular muscle, the pacing site most likely to allow that wavefront to depolarize as much ventricular muscle as possible is the one farthest from the Purkinje network.  For pacing from that site to also be able to enter the excitable gap and continuously reset (entrain) AVRT, it should be close to the ventricular insertion of the AP.  Basal ventricular sites close to the AP are therefore most likely to result in manifest entrainment. Indeed, when pacing very close to the ventricular insertion of the AP, the entrained QRS complex may be identical to that of the native tachycardia; this is called entrainment with concealed fusion (Figure 6).

***Teaching Point #8:*** * Concealed entrainment (QRS complex is that of a paced beat) is different from entrainment with concealed fusion (QRS complex is that of the native tachycardia). This subtle distinction in nomenclature is commonly overlooked (an error I have been guilty of as well [11]).*

***Teaching Point #9:*** * During orthodromic AVRT, the overdrive ventricular pacing site most likely to result in entrainment with fusion is at the base, near the insertion of the AP. Thus, entrainment with concealed fusion indicates that the pacing site is close to the ventricular insertion of the AP and can be used to map the AP [11].*

So far, we have been discussing manifest entrainment as it relates to the QRS complex morphology. However, evidence of fusion may also be apparent in intracardiac recordings. For instance, if during entrainment of orthodromic AVRT by overdrive pacing from the basal ventricle adjacent to the AP, the QRS complex morphology is that of a paced beat (concealed entrainment), one might be fortunate enough to see an orthodromically captured His potential preceding or immediately after the pacing stimulus (Figure 2,3,6). This His potential is proof that the orthodromic wavefront from the previous beat has reached the His bundle and will certainly collide with the stimulated antidromic wavefront either in the distal conduction system or just distal to that (but not within enough ventricular myocardium to alter the QRS complex morphology because it was described as that of a paced beat).

***Teaching Point #10:*** * An orthodromically captured His (or right bundle) potential can be considered evidence of fusion, indicating that orthodromic AVRT is present, even if the QRS complex morphlogy during pacing is that of a paced beat.*

**Figure 7:**
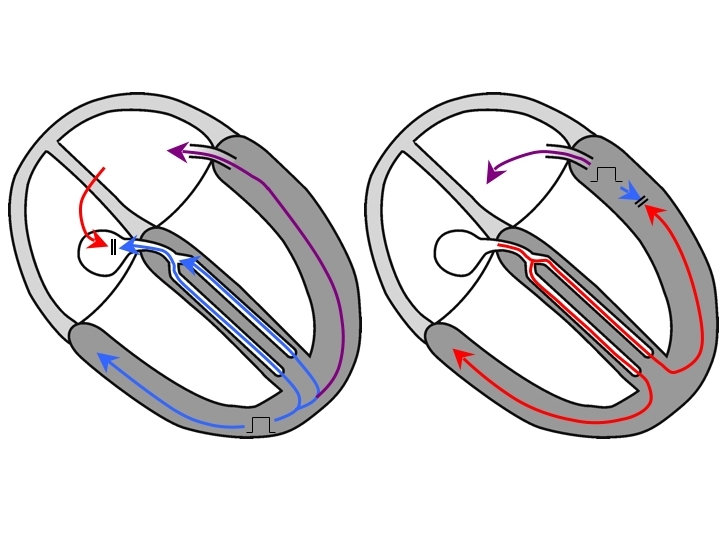
Entrainment of orthodromic AVRT employing a left free wall AP is depicted by pacing from the RV apex (left) and the basal LV close to the AP (right). Pacing from the RV apex must be delivered early enough that is has time to travel to and reach the AP so as to advance atrial timing and reset the tachycardia. Accordingly, the QRS complex will be that of a paced beat, and collision of the antidromic wavefront (blue) with the orthodromic wavefront fro the previous beat (red) will occur in the proximal AV conduction system (concealed entrainment). When the pacing site is close to the AP, the orthodromic wavefront from the previous beat can be allowed to exit the His-Purkinje and depolarize a significant amount of ventricular muscle before colliding with the stimulated antidromic wavefront. Thus, evidence of fusion is more likely to be present when the pacing site is close to the AP. Also, SA-VA and PPI-CL differences should be smaller after entrainment by pacing from a basal site than an apical one, enhancing the difference compared to entrainment of AVNRT circuits. AV=atrioventricular; AVRT=AV reciprocating tachycardia; AP=accessory pathway; RV=right ventricle; LV=left ventricle

In our experience, employing both QRS complex morphology and intracardiac recordings, fusion during entrainment of orthodromic AVRT is appreciable in about 50% of cases after pacing from the RVA and in about 75% of cases after pacing from the basal ventricle adjacent to the AP. Manifest entrainment is least likely to occur during entrainment of orthodromic AVRT employing a left sided AP by pacing from the RVA. The stimulated orthodromic wavefront must be delivered early enough that it can reach the left sided AP and enter the excitable gap of the tachycardia - so early that the paced wavefront will depolarize all of the ventricular myocardium and collision with the orthodromic wavefront from the preceding beat occurs in the proximal AV conduction system (Figure 7).

## When only concealed entrainment is possible

Further information is required to differentiate AVNRT from orthodromic AVRT when overdrive pacing (including from a basal ventricular site close to the earliest atrial activation) only results in concealed entrainment followed by an A-V response. If the septal VA interval during SVT < 70 ms, then a diagnosis of typical AVNRT can be made [4]^B^. When the septal VA > 70 ms, if the atrial activation sequence is eccentric, the most likely diagnosis is AVRT, though AVNRT with eccentric atrial activation is well recognized now [12-16]. When the septal VA > 70 ms and the atrial activation sequence is concentric, numerous other features and maneuvers have been suggested [17-20], one of which involves information already available in the tracings of concealed entrainment [21], and builds upon the principles so far reviewed.

## The PPI-TCL difference

The post pacing interval is the time required for the last stimulated orthodromic wavefront to reach the excitable gap of a circuit, travel around the circuit, and return to the pacing site. If the pacing site is in the circuit, then the PPI= tachycardia CL (TCL). The farther a pacing site is from a circuit, the greater the PPI-TCL difference will be. Because the RVA is close to orthodromic AVRT circuits involving right sided or septal APs, yet relatively far from AVNRT circuits, the PPI-TCL difference after entrainment from the RVA can be used to distinguish AVNRT from AVRT (particularly when atrial activation is concentric) (Figure 7). A PPI-TCL difference > 115 ms is consistent with AVNRT, while a PPI-TCL difference < 115 ms is consistent with AVRT [21].

A relatively common phenomenon encountered during entrainment of orthodromic AVRT by ventricular pacing is prolongation of the AH interval component of the A-V response due to conduction slowing through the AV node. This occurs because of the decremental conduction properties of the AV node (after all, the atrium, and hence the input to the AV node, is accelerated during entrainment). In cases where dual AV node physiology is present, it is also possible that the pacing CL during entrainment encroaches upon the refractory period of the fast AV node pathway such that the A-V response employs the slow AV node pathway [22]. Whether the prolonged AH interval on the last entrained beat is due to either of these factors, it will contribute to prolongation of the PPI that is not reflective of the distance of the pacing site from the circuit. Thus, PPI-TCL differences obtained after entrainment of orthodromic AVRT employing a septal AP can actually overlap with those observed after entrainment of AVNRT. For this reason, the corrected PPI-TCL difference (cPPI-TCL) is preferred [23]. The cPPI-TCL is determined by subtracting the increase in the first return AH interval (ie. first return AH interval - AH interval prior to entrainment) from the PPI-TCL (Figure 2,3,6). If a His potential is not recorded, assuming the HV interval remains constant, the correction can accommodate the increase in the first return AV interval compared to the AV interval in SVT.

***Teaching Point #11:*** * A cPPI-TCL > 110 ms is consistent with AVNRT, while a cPPI-TCL < 110 ms is consistent with AVRT employing a non-left sided AP.*

 A cPPI-TCL > 110 ms can occur with AVRT employing a left sided AP [23] simply because the RVA pacing site is far from such a circuit. During a long RP interval SVT, a cPPI-TCL > 110 ms should also prompt consideration of orthodromic AVRT employing a slowly conducting AP with decremental conduction properties.^C^

## The SA-VA difference

During orthodromic AVRT and during entrainment of orthodromic AVRT by ventricular pacing, the ventricle and atrium are activated in series. In contrast, during AVNRT, the ventricle and atrium are activated in parallel, while during entrainment of AVNRT, they are forced in series. Therefore, if the difference between the VA interval during entrainment and SVT is considered, it ought to be longer for AVNRT than for AVRT. The VA interval during entrainment is measured from the pacing stimulus to the atrial electrogram (SA) (Figure 2,3,6).

***Teaching Point # 12:*** * SA-VA differences < 85 ms are consistent with AVRT, while SA-VA differences > 85 ms are consistent with AVNRT [21].*

The SA-VA difference is not subject to decremental conduction through the AV node during the A-V response. However, the SA-VA difference could be > 85 ms if the pacing site is far from the operative AP (for instance, during entrainment by pacing from the RVA during orthodromic AVRT employing a left sided AP) or if the AP has decremental conduction properties (as might be encountered during a long RP interval SVT). Also, SA-VA differences have tended to dichotomize patients with AVNRT and AVRT less well than PPI-TCL and cPPI-TCL differences [21,23].

***Teaching Point #13:*** * the cPPI-TCL and SA-VA differences may be unreliable during SVTs with marked spontaneous beat-to-beat variation in TCL (>40 ms).*

## The importance of the pacing site

We have already seen that basal pacing sites close to the operative AP are more likely to produce manifest entrainment of orthodromic AVRT. A basal pacing site near the earliest atrial activation is also both farther from an AVNRT circuit than an apical pacing site would be, and closer to the AP employed in an AVRT circuit than an apical pacing site would be. Thus, the SA-VA and cPPI-TCL differences between AVNRT and orthodromic AVRT obtained after pacing from a basal site near the earliest atrial activation are likely to be greater than those differences obtained after pacing from the RVA, while the discriminant values should not change [24] (Figure 4 and 7). The basal anterior septum ought to be avoided as a pacing site because direct capture of the His or right bundle may occur. (While intentional para-Hisian entrainment can be diagnostically useful during SVT [20] as it exploits the anatomic differences between AVNRT and AVRT circuits in much the same way that entrainment by basal ventricular pacing near the earliest atrial activation does, it remains beyond the scope of this article.)

***Teaching Point#14:*** * If SA-VA and/or cPPI-TCL differences are borderline or discrepant after entrainment by pacing from the RV apex, entrainment from a basal ventricular site near the earliest atrial activation should be performed. Basal pacing may produce fusion (which would prove that AVRT is present) or clarify the diagnosis based on SA-VA and cPPI-TCL differences.*

**Figure 8:**
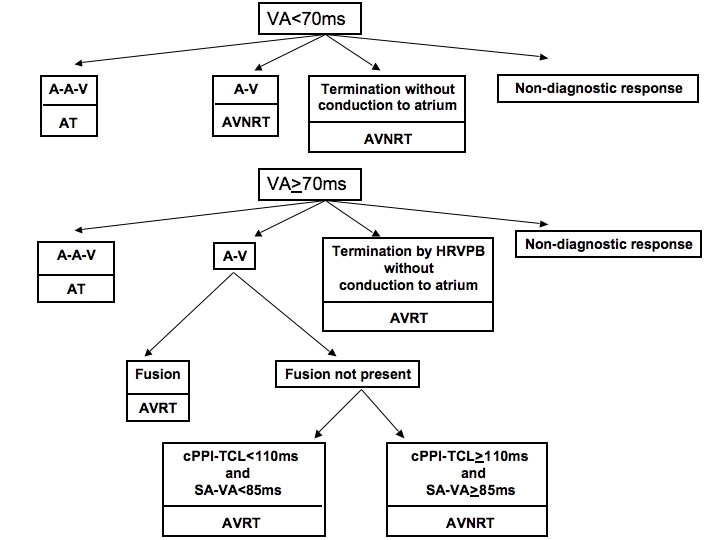
A proposed algorithm to arrive at a diagnosis for regular sustained SVT based on the results of overdrive ventricular pacing. Non-diagnostic responses may contain partial diagnostic information and  include (i) termination with conduction to the atria, (ii) termination of SVT with septal VA ≥ 70 ms by a VPB prior to His bundle refractoriness that does not conduct to the atrium (excludes AT), and (iii) dissociation of the ventricles from the tachycardia (excludes AVRT). A=atrium; V=ventricle; AT=atrial tachycardia; AVNRT=atrioventricular node reentry tachycardia; AVRT=atrioventricular reciprocating tachycardia; S=stimulus; PPI=post pacing interval; TCL=tachycardia cycle length; HRVPB=His refractory ventricular premature beat.

In our experience, overdrive ventricular pacing including a basal site near the earliest atrial activation provides a definitive SVT diagnosis in over 90% of regular sustained SVT's by assessing the post pacing atrial-ventricular response relationship, the presence of fusion, and the SA-VA and cPPI-TCL differences (Figure 8). This should hold true for all types of SVT including AVRT employing left sided AP's. In these cases, where the main differential diagnosis includes the rare circumstance of AVNRT employing a left atrionodal exit, the basal LV can sometimes be captured by pacing a branch of the CS. Distinguishing these 2 tachycardias is important because AVNRT with a left atrionodal exit can often be ablated in the usual location at the base of Koch's triangle, avoiding the need for ablation in the systemic circulation [16]. It is noteworthy that if only the RV apex is used for pacing, AVNRT may not be distinguishable from AVRT employing a left sided AP because the RVA is far from both of these circuits (so entrainment will likely be concealed and the cPPI-TCL and SA-VA differences may be long in either case).

As discussed elsewhere [25], overdrive ventricular pacing is also of diagnostic value in determining whether a pre-excited tachycardia involves a bystander AP or if the AP is participating in the tachycardia mechanism. In these circumstances, pacing from the RV apex commonly produces fusion [8] since the stimulated antidromic wavefront originates far from where the orthodromic wavefront from the previous beat depolarizes ventricular muscle, namely, at the basal insertion of the AP into ventricular muscle. Fusion would not be expected during entrainment of antidromic AVRT employing a long atriofascicular AP because these tend to insert into or very close to the distal conduction system. Even when entrainment is concealed, the PPI-TCL and SA-VA values may be of diagnostic value [25].

## Atrial Overdrive Pacing

Overdrive ventricular pacing is in part diagnostically useful because the ventricle is only a component of AP mediated SVT circuits. Because atrial muscle is a required component of AT, AVRT, and probably AVNRT (while that debate is ongoing, it is at least fair to say that AVNRT with block to the atrium is extremely rare, and even in those instances, it is difficult to exclude a protected component of atrial tissue), atrial overdrive pacing doesn't have the potential to differentiate among SVT mechanisms in a similar fashion. For instance, fusion due to wavefront collision in atrial muscle during entrainment of SVT by atrial pacing can occur during a reentrant atrial tachycardia or AVRT [8]. However, there are a few instances where atrial overdrive pacing can be diagnostically useful.

The differentiation of a focal junctional or 'His bundle' tachycardia from typical AVNRT can sometimes be challenging. Overdrive ventricular pacing would be expected to produce an A-V response in either case [3]. Entrainment of AVNRT by atrial pacing can be the key diagnostic maneuver: the demonstration of a long AH interval between the last paced atrial beat and the last entrained ventricular electrogram should identify the tachycardia as AVNRT because this observation demonstrates antegrade conduction through a slow AV node pathway.

Likewise, the differentiation of AVNRT from a septal atrial tachycardia can be difficult, particularly when ventricular overdrive pacing only dissociates the ventricles. Because activation of the atria and ventricles are usually linked in AVNRT, and not mechanistically related in the case of AT, a consistent post pacing VA interval after entrainment by pacing from multiple atrial sites is consistent with AVNRT. Alternatively, variability in the post pacing VA interval after atrial overdrive pacing from multiple distant atrial sites would be expected in the case of an atrial tachycardia because the timing of the first return atrial impulse will depend on the proximity of the pacing site to the origin of the AT [26], and not on the first return ventricular beat. Maruyama et al. have reported that the maximal difference in post pacing VA intervals after atrial overdrive pacing at 3 sites was < 14 ms for AVNRT and > 14 ms for septal AT [5]. This interesting small study should be externally validated.

## Conclusion

Entrainment is the repeated resetting of a reentrant circuit by a pacing train. All 4 of the criteria for entrainment (including the first 2 criteria, which we have explored as they pertain to AVRT and AVNRT circuits) simply demonstrate evidence that the following wavefronts exist: (i) the stimulated orthodromic wavefront that resets the circuit, (ii) the stimulated antidromic wavefront and (iii) the orthodromic wavefront from the preceding beat. A triggered or automatic focal arrhythmia cannot explain these phenomena, so the demonstration of the criteria for entrainment is evidence that the tachycardia mechanism is reentry. We have also seen that concealed entrainment implies the absence of demonstrable evidence of these wavefronts, as during entrainment of AVNRT. Finally, we have explored how these principles, which have most commonly been applied to elucidating unknown atrial and ventricular circuits, can be used to determine the mechanism of SVT by employing a single pacing maneuver and a simple algorithm.

## Footnotes

A This statement is true in the absence of a "bystander" AP. In the case of AT or AVNRT occurring in a patient who also has an AP, manifest entrainment by ventricular pacing proves that a sustainable AVRT circuit is indeed present, so that such an AP would not really be a "bystander" at all. Such an exceptional circumstance would require a double tachycardia or a double loop tachycardia where one of the tachycardias is orthodromic AVRT. Ablation of the AP would ultimately be required both clinically and to unmask the second tachycardia mechanism.

B In virtually all such cases, a diagnosis of typical AVNRT can be made. However, 2 extremely rare SVTs, namely, orthodromic nodoventricular or nodofascicular reentry tachycardia, are theoretically possible. The clue to one of these tachycardias in this situation would be SA-VA and cPPI-TCL values in keeping with AVRT, and in the case of nodoventricular tachycardia, manifest fusion or entrainment with concealed fusion [11].

C In these challenging situations, fusion during entrainment by ventricular pacing and delay of atrial timing by a His-refractory VPB should be considered proof of the involvement of an AP regardless of the PPI-TCL value.
